# Bereavement support guidelines for caregivers in palliative care: a scoping review

**DOI:** 10.3389/fpsyg.2025.1541783

**Published:** 2025-04-07

**Authors:** Alexandra Coelho, Sara Albuquerque, David Dias Neto

**Affiliations:** ^1^APPsyCI - Applied Psychology Research Center Capabilities & Inclusion; ISPA - Instituto Universitário, Lisbon, Portugal; ^2^Psylab, Faculty of Medicine, University of Lisbon, Lisbon, Portugal; ^3^HEI-Lab: Digital Human-Environment Interaction Labs, Lusófona University, Lisbon, Portugal

**Keywords:** bereavement, grief, palliative care, guidelines, clinical recommendations, family caregivers

## Abstract

**Background:**

Palliative care teams’ support practices for bereavement vary substantially. Clinical guidelines are needed to promote concerted, evidence-based intervention. The goal of the present study is to identify and synthesize the principles and clinical guidelines that ensure best practices in bereavement support for family caregivers accompanied in palliative care.

**Methods:**

A scoping review was conducted based on a systematic search of articles in academic databases (EBSCO, PsycINFO, PubMed, Web of Science, Psychology and Behavioral Sciences Collection, Scopus) and Google (2010–2024). The review included articles focused on the principles, guidelines, and clinical recommendations for bereavement support for adult family caregivers in palliative care. Quality appraisal of guidelines was conducted using the AGREE II instrument.

**Results:**

Of the 1,489 references identified, 20 documents were included, mostly governmental or institutional norms and clinical guidelines from gray literature. Quality appraisal revealed gaps in evidence selection, resource implications, updates and monitoring criteria. Eight fundamental principles were identified, from which several clinical guidelines were derived, organized according to the moments of assessment and intervention throughout the bereavement process, including pre and post-death period: (1) organizing support for the family caregiver; (2) assessing needs and establishing a care plan; (3) ensuring information and support for the family caregiver; (4) preparing for death; (5) support at the time of death; and (6) bereavement support post-death. In addition to universal support and information measures, regular assessment procedures should be adopted for timely referrals based on individual needs.

**Discussion:**

These guidelines cover the temporal variation of care and the multidimensional and multiple-actor nature of palliative care. Implementing these guidelines and evaluating their impact will allow for the standardization of best practices and improve the quality of bereavement support in palliative care.

## 1 Introduction

Grief is a common and natural response to the loss of someone significant. It typically involves a period of mourning and adaptation to the absence, with feelings of sadness, loneliness, and longing. While most individuals adjust to the loss, some bereaved experience symptoms of Prolonged Grief Disorder (PGD), characterized by intense yearning and disruptive emotional pain, pervasive preoccupation with the deceased, difficulty in accepting the loss, identity disruption and loss of meaning in life, often lasting for an extended period and beyond what is culturally expected (Prigerson et al., 2009). Due to its persistent and debilitating nature, this condition demands clinical intervention. According to cross-nation studies, its prevalence rates reach 10–13% of bereaved people (Comtesse et al., 2024; Lundorff et al., 2017).

Although PGD was recently recognized as an independent mental disorder in ICD11 (World Health Organization, 2018) and DSM-V-Tr (American Psychiatric Association, 2022), it often co-occurs with other mental health conditions, such as Depression, Anxiety, and Post-traumatic Stress Disorder, further complicating its diagnosis and treatment (Komischke-Konnerup et al., 2021; Rheingold et al., 2024). These overlapping disorders can exacerbate the emotional pain and distress experienced by individuals suffering from PGD. Additionally, PGD is associated with severe consequences for an individual’s wellbeing, including suicidal ideation (Sekowski and Prigerson, 2022), functional impairment (Nielsen et al., 2020), and a significant reduction in overall quality of life (Maccallum and Bryant, 2020). The impact of PGD extends beyond mental health, leading to sleep disturbances (Lancel et al., 2020), cardiovascular problems (Palitsky et al., 2023) and an elevated risk of mortality, partly because of risky behaviors (Hiyoshi et al., 2022; Prigerson et al., 2009).

Family caregivers have been identified as a risk group for the development of PGD. Throughout the disorder trajectory, family caregivers experience various types of losses related to the functional decline and degradation of the patient’s image (Coelho et al., 2020). These losses give rise to a process of anticipatory grief, which is often adaptive, as it functions as a preparation against the news of sudden death. However, it can also be a source of great distress, thus becoming predictive of difficulties adapting to the loss (Nielsen et al., 2016). Other situational and risk factors associated with the caregiver’s poor bereavement outcomes include the family member’s nervousness and stress (Liljeroos et al., 2022), less preparedness for the caregiving role, greater impact of caring on schedule, relationship strain, lack of social support, lower active coping mechanisms, greater impact on caregiver’s health (Miller et al., 2018) and poor family functioning (Thomas et al., 2014). Besides that, pre-existing psychopathology (Ribera-Asensi et al., 2024), insecure attachment styles, such as anxiety and avoidance, and high dependency on the deceased also increase the risk of prolonged grief (Mason et al., 2020; Miller et al., 2018).

The prevalence of Prolonged Grief Disorder (PGD) among caregivers in Palliative Care settings is notably high, with studies indicating that up to 11.3% of caregivers experience PGD symptoms 11 months after the death of the patient (Thomas et al., 2014). Furthermore, a substantial proportion of caregivers continue to display sub-threshold symptoms of PGD even years after the loss, with one study showing that 14% of caregivers still experienced significant distress at 37 months post-bereavement (Zordan et al., 2019). These findings underscore the critical need for long-term bereavement care to address the ongoing challenges that caregivers face in adapting to the loss.

Unfortunately, bereavement support is not always effectively targeted at those who require it the most. Research suggests that many individuals who are most vulnerable to prolonged grief do not receive the necessary help (Lichtenthal et al., 2011), pointing to deficiencies in current screening methods used to identify those at greatest risk (Morris et al., 2019). Moreover, while it is evident that intervention during complex grief situations can be highly beneficial, the application of generalized, one-size-fits-all approaches to bereavement support is counterproductive. Specialized, tailored interventions are more effective in addressing the specific needs of individuals experiencing complicated grief rather than relying on universal interventions for all bereaved individuals (Currier et al., 2008). This highlights the importance of improving screening procedures and customizing support to ensure that those at higher risk of developing PGD receive appropriate and effective care.

Considering the need to adapt resources and intervention measures to the individual needs of bereaved people, the National Institute for Health Clinical Excellence (2004) proposes the organization of bereavement care according to a three-level approach. First, universal interventions (primary prevention) are directed at all bereaved people with basic support needs (normal grief). They are carried out mainly by the community, health professionals, and senior social service professionals without specific training in bereavement. These include disseminating informative materials and literature on bereavement (at various stages of development), telephone contact and bereavement letters to acknowledge death, awareness-raising and educational actions, and memorial services. Second, selective interventions (secondary prevention) are directed at people with intermediate support needs in bereavement (with mild or moderate symptoms or at risk of developing PGD). They are carried out by mental health professionals with intermediate training in bereavement. It involves telephone support, home visits, peer group referrals, and individual support by volunteers or non-specialized professionals. Third, indicative interventions (tertiary prevention) are directed at people with complex support needs in bereavement (with symptoms of PGD). They are carried out by mental health professionals with advanced training in bereavement. It involves individual or group follow-up in bereavement counseling, which may be complemented by the intervention of trained volunteers.

Support for bereaved family members is recognized as one of the pillars of palliative care. This holistic approach provides relief from pain and other symptoms while also addressing the psychological, social, and spiritual needs of patients and their families during the illness trajectory and after the death of a loved one. By addressing grief in a compassionate and structured manner it provides a privileged context for early detection and preventive intervention for the most vulnerable individuals, allowing continuous, systematic assessment and follow-up from the phase preceding death (Neimeyer, 2020). The application of the public health model to bereavement support in palliative care emphasizes a population-based approach to addressing the needs of families and individuals coping with loss (Aoun et al., 2012). This model recognizes grief as a universal experience affecting the immediate family and the broader community. Also, it encourages the systematic collection of data to monitor trends in bereavement and identify those at higher risk for complicated grief, ensuring that appropriate interventions are provided. Ultimately, applying a public health approach to bereavement support in palliative care enhances the ability to reach a broader population, fostering a sense of collective support and resilience during times of loss.

Nevertheless, there is evidence of unmet psychosocial and spiritual needs of family members, particularly in preparing for and confronting the proximity of the death of a significant person and support in bereavement (Hashemi et al., 2018). Also, Breen et al. (2014) argues that while theoretical frameworks advocate for comprehensive support systems for families, the reality in clinical settings often falls short. Katz et al. (2023) found low rates of grief and bereavement support pre- and post-death for families, indicating a systemic issue in providing necessary care. Additionally, Naef et al. (2020) highlight that the adoption of structured follow-up care has been notoriously low, despite an agreed-upon mandate to engage with and care for family members at the end-of-life. Together these studies highlight a significant gap between what is advocated in the literature and clinical practice. Therefore, clinical guidelines that promote concerted and evidence-based action to improve bereavement support in palliative care are needed (e.g., Morris and Sannes, 2020).

For clarification, by principles, we mean the general norms of conduct that guide good clinical practice, thus underlying the clinical guidelines. Clinical guidelines, also known as standards, consist of specific recommendations developed based on the best empirical evidence on how to proceed in clinical practice, thus supporting professionals in decision-making about diagnosis and treatment [Agency for Healthcare Research and Quality (AHRQ), 2025].

### 1.1 Present study

While bereavement support is a vital aspect of palliative care, there is often a gap between recommended practices and the actual support provided by palliative care services. Many services adopt a generic approach, which may not adequately address the specific needs of bereaved individuals. Barriers to effective bereavement support include insufficient resources, lack of systematic application, and inadequate assessment of bereavement risk (Aoun et al., 2017). In this study, we conduct a scoping review of the literature to identify the principles and clinical guidelines for providing bereavement support to adult family caregivers involved in palliative care, throughout the end-of-life process, death and the post-death bereavement period. The following question guided this study: According to recent literature, what are the existing principles and guidelines established for bereavement support for adult family caregivers in palliative care?

## 2 Methods

### 2.1 Design

A scoping review of the literature was conducted to map the existing guidelines on the scientific literature. This method is particularly suited when there is little evidence to provide direction and fill the gaps between research and practice (Levac et al., 2010). The review was conducted following the methodology proposed by Arksey and O'Malley (2005), which provides a flexible framework to map the evidence, involving five distinct phases: (1) Identification of the research question; (2) Identification of relevant studies; (3) Selection of studies; (4) Mapping of data; (5) Bringing together, summarizing, and presenting the results. Consultation with stakeholders or experts validated results and provided further insights. Additionally, a systematic quality assessment of guidelines was conducted to identify strengths and weaknesses in the guidelines, such as the clarity of recommendations, the transparency of evidence selection, and the involvement of stakeholders in the development process. This process not only enhances the reliability of the findings, but also supports the identification of gaps in the existing guidelines, guiding future research and informing practice more effectively.

### 2.2 Eligibility criteria

Documents were eligible if they met the following inclusion criteria: (1) original studies or reports of principles, guidelines, or recommendations for clinical practice in bereavement support; (2) targeted to family caregivers of adult patients in palliative care or in a situation of advanced chronic illness; (3) published in English, Portuguese, or Spanish; (4) developed by a government organization, NGO commissioned by State/Federal Government, a National Professional Association or a group of clinicians or experts on the field. As exclusion criteria, we considered: (1) guidelines targeted to other populations (e.g., neonatology, pediatrics, loss of minor children, death by suicide, sudden death); (2) studies on the prevalence of PGD, comorbidity, risk factors, or specific mechanisms of grief; (3) studies validating bereavement assessment instruments; (4) studies focused on the organization of bereavement services; (5) studies on the satisfaction and quality of end-of-life care; (6) studies on the evaluation of the quality of guidelines; (7) articles without full-text access; (8) created by a single author or as a part of a dissertation. The first author (AC) researched the databases, downloaded the articles into Mendeley to remove duplicates and initially selected the articles based on the title and abstract. Then, two authors (AC and SA) independently verify the accuracy and eligibility of the full-text articles. Disagreements in the selection process were resolved through discussion. Reasons for exclusion were registered in an Excel document.

### 2.3 Search strategy

The search, conducted in September–October 2024, included literature published in the last 14 years (2010–2024). This time limit was set to capture the most recent literature. The academic databases EBSCO, PsycINFO, PubMed, Web of Science, Psychology and Behavioral Sciences Collection, and Scopus were used with the following search terms: “guidelines” OR “practice guideline” OR “clinical practice guideline” OR “recommendation” OR “consensus” AND “grief” OR “griev*” OR “loss” OR “bereav*” OR “mourn*” AND “palliative care” OR “terminal care” OR “end of life care” OR “hospice care.” The Google and Google Scholar search engines were also used with the same keywords to access gray literature. Finally, a manual search was conducted based on the bibliographic references from a previous literature review (Kent et al., 2020).

### 2.4 Data charting

Data regarding the type of document, title, authors, year of publication, location, target population, method, and results were collected and organized in tables. This resulted in creating a descriptive table of the main characteristics of the studies (title, authors, country, year, and target population).

### 2.5 Data analysis

This phase refers to the qualitative and quantitative analysis of the results. Regarding the qualitative analysis, the main results (principles and clinical guidelines) were thematically analysed according to the method outlined by Arksey and O’Malley (2005). First, the first author (AC) coded the extracted data line-by-line to create an initial thematic framework that described the approaches and best practices in supporting family caregivers throughout the bereavement process, from the admission in palliative to the post-death bereavement period. Codes were inductively developed based on key data extraction and refined into broader concepts when overlaps occurred, generating initial themes. Then, a group of experts in grief and bereavement in palliative care was selected (five clinical psychologists and two social workers), and they were provided with materials, including the thematic framework and background information on the data extraction process. The goal was to gather feedback on the framework’s accuracy and completeness. The experts reviewed and discussed the themes, offering insights and suggesting modifications. The discussion was structured to focus on refining and enhancing the identified themes. After the discussion, AC synthesized the feedback, reviewed the framework accordingly, and shared it with the panel for a final review. Then, the research team revised the coded data and the full-text articles to add detail and finalize the analytical thematic framework.

Regarding the quantitative analysis, a systematic evaluation of the quality of each guideline was conducted with the Appraisal of Guidelines, Research and Evaluation (AGREE II) checklist (Brouwers et al., 2010). A summary of the AGREE II structure and a detailed list of items within each scoring domain are displayed in Table 1. This is a widely used tool in health-related fields, which mainly assesses the process of developing guidelines rather than their content. The AGREE II is composed of six domains: Scope and Purpose (items 1–3), Stakeholder Involvement (items 4–6), Rigor of Development (items 7–14), Clarity of Presentation (items 15–17), Applicability (items 18–21), and Editorial Independence (items 22–23). Each item is evaluated according to a 7-point scale ranging from 1 (strongly disagree, indicating no relevant information is provided) to 7 (strongly agree, indicating the quality of reporting is exceptional). The first two authors (AC and SA) independently rated each guideline across the six domains of the AGREE II checklist. Each item within the domains was rated on a 7-point scale ranging from 1 (strongly disagree) to 7 (strongly agree), reflecting their assessment of how well the guideline met the criteria outlined in the tool. Interrater reliability (agreement between the two reviewers’ item scores) was calculated using the (two-way mixed) intraclass correlation coefficient (ICC) with SPSS software (SPSS version 29.0; IBM Corporation, 2023). Agreement was described as follows: <0.20 poor; 0.21–0.40 fair; 0.41–0.60 moderate; 0.61–0.80 good; 0.81–1.00 very good. The domain scores were determined by summing the item scores within each domain provided by both reviewers and converting the total into a percentage of the maximum possible score for that domain. Additionally, the mean and standard deviation were calculated to determine a “total domain score” for each practice guideline, resulting in an “overall quality rating,” classified as good (80% or higher), acceptable (60–79%), low (40–59%), or very low (below 40%).

**Table 1:** Summary of AGREE II structure and detailed list of items within each scoring domain.

Domain name	Item	Feature to be evaluated
Scope and purpose	1	The overall objective(s) of the guideline is (are) specifically described
	2	The health question(s) covered by the guideline is (are) specifically described
	3	The population to whom the guideline is meant to apply is specifically described
Stakeholder involvement	4	The guideline development group includes individuals from all the relevant professional groups
	5	The views and preferences of the target population (patients, public, etc.) have been sought
	6	The target users of the guideline are clearly defined
Rigor of development	7	Systematic methods were used to search for evidence
	8	The criteria for selecting the evidence are clearly described
	9	The strengths and limitations of the body of evidence are clearly described
	10	The methods for formulating the recommendations are clearly described
	11	The health benefits, side effects, and risks are considered in formulating the recommendations
	12	There is an explicit link between the recommendations and the supporting evidence
	13	The guideline has been externally reviewed by experts prior to its publication
	14	A procedure for updating the guideline is provided
Clarity of Presentation	15	The recommendations are specific and unambiguous
	16	The different options for management of the condition or health issue are clearly presented
	17	Key recommendations are easily identifiable
Applicability	18	The guideline describes facilitators and barriers to its application
	19	The guideline provides advice and/or tools on how the recommendations can be put into practice
	20	The potential resource implications of applying the recommendations have been considered
	21	The guideline presents monitoring and/or auditing criteria
Editorial independence	22	The views of the funding body have not influenced the content of the guideline
	23	Competing interests of guideline development group members have been recorded and addressed

## 3 Results

**Figure 1:**
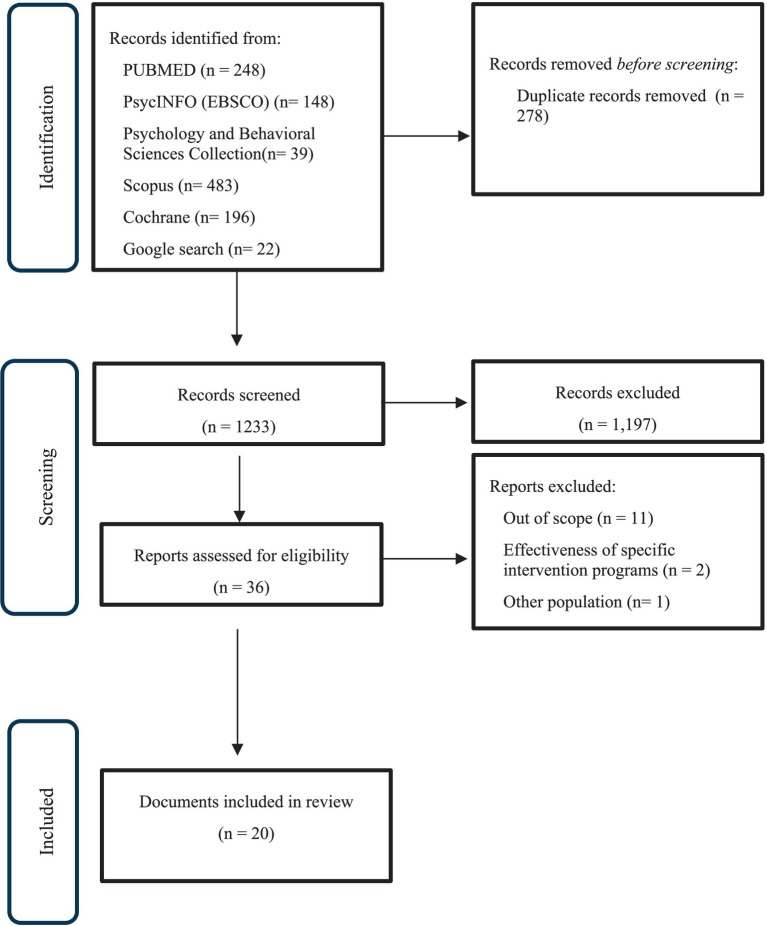
Flowchart PRISMA of search results.

A total of 1,489 documents were identified by searching academic databases and Google. After excluding duplicate records, the first author made an initial selection based on reading the titles and abstracts (*n* = 120), resulting in the exclusion of 1,173 records that did not meet the inclusion criteria. The remaining 38 documents were read in full, with 17 being excluded for the following reasons: out of scope (*n* = 11); studies evaluating the effectiveness of intervention in bereavement (*n* = 2); other population (*n* = 1); no access to the full article (*n* = 2). A total of 20 documents were included in the review, mostly from gray literature (*n* = 13). The selection process is described in the PRISMA flowchart (Figure 1).

Seven of the 20 documents included in the review (Table 2) were published as scientific articles; the others (*n* = 13) were government or institutional norms, including palliative care and bereavement support organizations. The clinical guidelines were developed in the following countries: Australia (*n* = 9), United States of America (*n* = 4), New Zealand (*n* = 2), Canada (*n* = 2), Ireland (*n* = 1), South Korea (*n* = 1) and Singapore (*n* = 1). For a detailed list of specific guidelines per country, please refer to Table 2, where each guideline matches the corresponding country of origin. All standards included were specifically oriented toward the population of caregivers in palliative and advanced cancer care.

**Table 2:** Guidelines and standards included in the review.

	Author, year	Title	Country	Target population
1	Hudson et al. (2010)	Clinical Practice Guidelines for the Psychosocial and Bereavement Support of Family Caregivers of Palliative Care Patients	Australia	Palliative care
2	SGVDH (2012)	Bereavement support standards for specialist palliative care services	Australia	Palliative care
3	Hudson et al. (2012)	Guidelines for the Psychosocial and Bereavement Support of Family Caregivers of Palliative Care Patients	Australia	Palliative care
4	NSPCIT (2014)	National Guidelines for Palliative Care	Singapore	Palliative care
5	NCCN (2014)	NCCN Clinical Practice Guidelines in Oncology (NCCN Guidelines)	USA	Palliative care
6	MDHBPC (2015)	Palliative Care Bereavement Support Guidelines	New Zealand	Palliative care
7	GRPCC (2016)	Bereavement Risk Screening and Management Guidelines	Australia	Palliative care
8	Philip et al. (2018)	A proposed framework of supportive and palliative care for people with high-grade glioma.	Australia	Palliative care
9	BCCPC (2017a, 2017b)	BC Inter-Professional Palliative Symptom Management Guidelines	Canada	Palliative care
10	BCCA (2017)	Palliative Care for the Patient with Incurable Cancer or Advanced Disease Part 3: Grief and Bereavement	Canada	Palliative care
11	MHW (2017)	Te Ara Whakapiri - Principles and guidance for the last days of life	New Zealand	End-of-life Care
12	Hudson et al. (2017)	Bereavement support standards and bereavement care pathway for quality palliative care.	Australia	Palliative care
13	Ferrell et al. (2018)	National Consensus Project Clinical Practice Guidelines for Quality Palliative Care	USA	Palliative care
14	PCA (2018)	National Palliative Care Standards	Australia	Palliative care
15	NHPCO (2018)	Standards of Practice for Hospice Programs	USA	Palliative care
16	Shin et al. (2020)	Clinical Practice Guideline for Care in the Last Days of Life	South Korea	Hospice and Palliative Care
17	Keegan et al. (2021)	EAPC Bereavement Task Force	Ireland	Palliative care
18	Morris and Sannes (2020)	Bereavement care for family caregivers of neuro-oncology patients	USA	Neuro-oncology
19	NSWACI (2024)	Clinical Principles for End-of-Life and Palliative Care	Australia	End-of-life Care
20	NSWACI (2024)	Clinical principles for specialist bereavement care in NSW	Australia	Palliative care

The results of the quality appraisal of guidelines using AGREE II are presented in Table 3. The highest scores were observed in the categories of “Scope and Purpose,” “Clarity of Presentation,” and “Stakeholder involvement,” showing that guidelines are well-structured, with clear objectives focused on improving psychosocial and bereavement support for family caregivers of palliative care patients. They effectively identified their target audience, namely family caregivers, and were developed by a multidisciplinary team in collaboration with key stakeholders. Additionally, the guidelines offer specific and unambiguous recommendations, accompanied by practical advice and tools to facilitate the implementation of bereavement support services. In contrast, the “Rigor of Development,” “Applicability,” and “Editorial Independence” categories showed lower domain scores. Most guidelines lack explicit criteria for selecting evidence and fail to clearly link recommendations to supporting evidence. Furthermore, they do not outline procedures for updating the guidelines or discuss the potential resource implications of implementing the recommendations. Lastly, monitoring or auditing criteria are not explicitly addressed, leaving gaps in assessing the guidelines’ effectiveness. Significant variability was evident in the scores, particularly in “Rigor of Development” and “Editorial Independence.” Most guidelines obtained a low overall quality rating; only two guidelines (Shin et al., 2020; NSWACI, 2024) were considered “Good.” Interrater reliability analysis showed very good agreement between the two reviewers for all guidelines (ICC range 0.84–0.99).

**Table 3:** Summary of domain scores for guidelines using AGREE II.

		Domain 1 Scope and purpose	Domain 2 Stakeholder involvement	Domain 3 Rigor of development	Domain 4 Clarity of presentation	Domain 5 Applicability	Domain 6 Editorial independence	Total domain score Mean (SD)	Overal quality rating^a^	ICC (95% CI)
1	Hudson et al. (2010)	100	91.60	36.25	97.22	12.50	0	56.26 (45.43)	Low	0.98 (0.95–0.99)
2	SGVDH (2012)	83.3	75.00	27.08	97.22	27.08	0	51.61 (38.73)	Low	0.97 (0.93–98)
3	Hudson et al. (2012)	100.0	97.20	84.37	97.22	43.75	0	70.42 (40.44)	Acceptable	0.98 (0.95–0.99)
4	NSPCIT (2014)	100	100.00	33.33	97.22	50.00	0	63.43 (42.25)	Acceptable	0.97 (0.94–0.99)
5	NCCN (2014)	100	75.00	84.37	100	39.58	29.17	71.35 (30.37)	Acceptable	0.91 (0.81–0.96)
6	MDHBPC (2015)	86.10	80.50	51.04	77.78	50	0	57.57 (32.13)	Low	0.94 (0.86–97)
7	GRPCC (2016)	100	100.00	63.54	97.22	60.42	0	70.20 (38.92)	Acceptable	0.96 (0.88–98)
8	Philip et al. (2018)	100	88.80	27.08	72.22	31.25	0	53.23 (39.53)	Low	0.99 (0.97–0.99)
9	BCCPC (2017a, 2017b)	100	47.20	17.71	100	33.33	0	49.71 (42.02)	Low	0.98 (0.96–0.99)
10	BCCA (2017)	94	47.20	15.63	97.22	37.50	0	48.66 (40.00)	Low	0.98 (0.96–0.99)
11	MHW (2017)	100	88.80	16.67	75.00	29.17	0	51.61 (41.62)	Low	0.98 (0.95–0.99)
12	Hudson et al. (2017)	100	69.40	15.63	88.89	33.33	0	51.21 (40.84)	Low	0.97 (0.94–0.99)
13	Ferrell et al. (2018)	100	83.30	15.63	27.78	16.67	0	40.56 (40.89)	Low	0.97 (0.93–0.98)
14	PCA (2018)	100	94.40	26.04	83.33	20.83	0	54.10 (43.37)	Low	0.98 (0.95–0.99)
15	NHPCO (2018)	94.40	75.00	19.79	94.44	52.08	0	55.95 (39.44)	Low	0.87 (0.63–95)
16	Shin et al. (2020)	100	83.30	83.33	97.22	70.83	83.33	86.34 (10.70)	Good	0.84 (0.44–94)
17	Keegan et al. (2021)	100	47.20	15.63	80.56	20.83	0	44.04 (39.40)	Low	0.97 (0.93–98)
18	Morris and Sannes (2020)	100	69.40	26.04	52.78	14.58	0	43.80 (37.37)	Low	0.99 (0.98–99)
19	NSWACI (2024)	91.60	50.00	83.33	100.00	85.42	29.17	73.25 (27.52)	Acceptable	0.92 (0.82–0.96)
20	NSWACI (2024)	100	100.00	88.54	97.22	79.17	29.17	82.35 (27.28)	Good	0.95 (0.79–0.98)
	Total domain scores Mean (SD)	97.49 (5.04)	78.17 (18.32)	41.55 (28.36)	86.53 (18.61)	40.42 (21.10)	8.54 (20.57)	58.78 (12.89)		

Data for each domain are expressed as a percentage of the maximum possible score (scale 1–7). ^a^Overal quality rating scores classified as good (80% or higher), acceptable (60–79%), low (40–59%), or very low (below 40%). SD, Standard Deviation; ICC, Intraclass Correlation Coefficient; CI, Confidence Interval.

Given that the majority were rated as “Low” or “Acceptable,” it was important to include these guidelines to ensure a comprehensive overview of the existing literature. The inclusion of lower-rated guidelines allows for a broader understanding of the current state of guidelines in the field, even though their quality may be suboptimal. This approach aligns with the goal of scoping reviews, which aim to map the breadth of available evidence.

### 3.1 Principles that guide support for caregivers

The definition of bereavement support principles serves as a foundational framework for developing guidelines with specific practice recommendations. These principles provide a theoretical and ethical basis, ensuring that care strategies are aligned with the needs of those experiencing grief. By grounding guidelines in these principles, practitioners are equipped with evidence-based, compassionate, and culturally sensitive approaches that address the complexities of bereavement. This alignment ensures consistency in care delivery while allowing flexibility to adapt to individual circumstances, ultimately enhancing the effectiveness and sensitivity of bereavement support interventions. Based on a thematic analysis of the documents, we propose eight principles to guide bereavement support for caregivers of palliative care patients.

1. Recognizing and responding to bereavement according to individual needs. Bereavement support should be individualized, sensitive and flexible, acknowledging and respecting each individual’s unique characteristics, needs, and preferences (MDHBPC, 2015; MHW, 2017; SGVDH, 2012). Bereavement should be understood as a normal response to the loss of a significant person, allowing individuals to adapt to a new reality. It is characterized by a range of emotional, physical, cognitive, behavioral, social, and economic reactions. Bereavement is influenced by individual, relational, social, spiritual, and cultural factors. Most individuals possess some resilience, i.e., a natural capacity to adapt and cope during periods of heightened stress and adversity. With the support of their family, friends, and established community networks, the bereaved are generally able to navigate challenges effectively and adjust to loss (MDHBPC, 2015; Keegan et al., 2021; Morris and Sannes, 2020). However, some individuals experience difficulties and may develop health problems (Keegan et al., 2021). Palliative care should advocate for policies that support bereaved individuals (Keegan et al., 2021). Family members and significant others of patients are eligible for bereavement services in palliative care settings. While the primary focus should be on the primary caregiver, support can be extended to other significant individuals (MDHBPC, 2015; SGVDH, 2012). Support should be tailored to individual needs, providing basic support to all bereaved individuals and additional care for those at risk (Keegan et al., 2021; Morris and Sannes, 2020). Healthcare professionals should be able to recognize when support needs exceed their capabilities and refer individuals to mental health specialists (Keegan et al., 2021; Morris and Sannes, 2020; NSWACI, 2024).
2. Accessible, equitable and culturally sensitive support. Bereavement support ought to be accessible, equitable and respectful of individual differences, including gender, age, socioeconomic status, cognitive abilities, sexual orientation, religion, culture and spirituality (MDHBPC, 2015; MHW, 2017; Morris and Sannes, 2020; NSWACI, 2024; SGVDH, 2012; SGVDH, 2012). Bereavement services must ensure cultural safety for culturally and linguistically diverse populations, individuals identifying as LGBTIQ+, and other priority groups. Access to bereavement services should be available to all who need it, irrespective of where the person died and whether the deceased was known to a palliative care service (MDHBPC, 2015; NSWACI, 2024). Support is expected to be readily available through phone, online, letter, domiciliary visits, or outpatient services (Ferrell et al., 2018; MDHBPC, 2015; MHW, 2017; SGVDH, 2012). Waiting times must be reasonable, ideally within five business days (MHW, 2017). Individuals should be informed about available local resources to make informed decisions and plan for their support needs (Keegan et al., 2021; NHPCO, 2018; PCA, 2018). Barriers to accessing specialized care should be minimized, especially considering the vulnerability and resistance often associated with addressing loss, particularly in the same physical context where end-of-life care and the death of the loved one occurred (MHW, 2017). Culturally appropriate referrals should involve coordination and collaboration with multicultural health workers. With the bereaved individual’s consent, this process may include detailed handovers to specialist bereavement services to uphold cultural safety within the receiving service (NSWACI, 2024).
3. Safety, privacy, confidentiality and respectful communication. Bereavement programs must adhere to ethical principles and ensure the privacy and confidentiality of bereaved individuals in compliance with data protection laws. Informed consent is required before providing any support, data sharing, or interventions, with clear communication about processes and options to drop out. Privacy and confidentiality are upheld through secure information storage, authorized access, and a physically private and feedback-friendly environment (Morris and Sannes, 2020; NSWACI, 2024). Communication with bereaved individuals must be sensitive, transparent, and honest, with shared decision-making, both before and after death (Hudson et al., 2010, 2012; MDHBPC, 2015; MHW, 2017; Morris and Sannes, 2020; NSPCIT, 2014). Information and resources about loss and grief ought to be provided routinely to families and carers before and after the death (PCA, 2018; NSWACI, 2024). Information needs to be presented in an accessible manner, tailored to the individual’s needs and at different time points. Written or audiovisual materials should be provided to enhance understanding (Hudson et al., 2010; Morris and Sannes, 2020; SGVDH, 2012). While healthcare professionals may suggest assessments and interventions, the ultimate decision-making authority rests with the bereaved individual. Sufficient time and information should be provided to enable informed choices. Before collecting information, oral consent must be obtained and documented (SGVDH, 2012).
4. Multidisciplinary assessment and ongoing emotional support. Healthcare professionals and volunteers should coordinate to ensure seamless delivery of services across different levels of support (Hudson et al., 2010; Keegan et al., 2021; MDHBPC, 2015; PCA, 2018). Ongoing assessment of the risk of complicated grief is essential, beginning at the onset of palliative care and continuing for several months after the loss, if necessary (Ferrell et al., 2018; NHPCO, 2018; Morris and Sannes, 2020; Keegan et al., 2021; NSWACI, 2024; PCA, 2018). Bereavement support should extend from the pre-death period to several months or years after death, with no time limit (Keegan et al., 2021; MDHBPC, 2015; NSWACI, 2024). Professionals must be vigilant for signs of complicated grief and potential mental health problems, referring individuals to specialized care as needed (Keegan et al., 2021; NHPCO, 2018; SGVDH, 2012). Seeking professional help should be normalized and encouraged (GRPCC, 2016).
5. Professional and volunteer development and self-care. Healthcare professionals and volunteers involved in palliative care are supposed to receive ongoing training to equip them for their role in providing bereavement support (Hudson et al., 2010, 2012; Keegan et al., 2021; MDHBPC, 2015; MHW, 2017; Morris and Sannes, 2020; SGVDH, 2012; NSWACI, 2024; PCA, 2018). Bereavement specialists should have advanced training and mandatory access to professional supervision (Keegan et al., 2021). Volunteers working with bereaved individuals should receive guidance from a bereavement specialist and have access to professional supervision (NHPCO, 2018). Administrative staff working within or associated with the bereavement service, whether through overarching organizations or service provision networks, must receive at least basic training in the core principles of grief and bereavement support strategies (NSWACI, 2024). It is recommended that palliative care services share evidence-based recommendations with other healthcare professionals (Keegan et al., 2021). Reflective practice, such as discussions and targeted courses, must be encouraged (NHPCO, 2018). Professionals working with death and bereavement are susceptible to vicarious trauma and burnout. Self-care practices, such as peer support and individual or group therapy, are recommended (Ferrell et al., 2018; GRPCC, 2016; Keegan et al., 2021; MHW, 2017; SGVDH, 2012). Employers have a plan for bereaved professionals (MDHBPC, 2015).
6. Community awareness and involvement. Palliative care services should promote the development of basic bereavement skills among other healthcare professionals and the community (Keegan et al., 2021; PCA, 2018). The impact of bereavement should be recognized and addressed through community awareness campaigns (NSWACI, 2024; SGVDH, 2012). The entire community, including schools, universities, social services, primary care, law enforcement, mental health services, and businesses, plays a role in supporting bereaved individuals. Bereavement programs should collaborate with a network of healthcare providers (Keegan et al., 2021; MHW, 2017; NSWACI, 2024; SGVDH, 2012).
7. Planning, evaluation, and research. Each service must develop its bereavement support protocol, outlining specific recommendations and defining team members’ roles (Keegan et al., 2021). A qualified professional should be appointed to coordinate bereavement support activities, i.e., tertiary qualifications in counseling, psychology or psychotherapy, social work, accreditation, membership or eligibility for membership of recognized associated professional bodies (Keegan et al., 2021; NSWACI, 2024). Services ought to be planned based on identified needs and regularly reevaluated. Palliative care services are recommended to allocate adequate human and material resources to bereavement support programs, including funding for training and supervision (Keegan et al., 2021; SGVDH, 2012). Bereavement services must engage staff with appropriate experience to offer counseling and support to social and cultural groups. If such expertise is unavailable, they actively establish partnerships with other service providers or organizations with the necessary experience (NSWACI, 2024). The quality of services should be monitored and continuously improved (Keegan et al., 2021; MDHBPC, 2015; MHW, 2017; NHPCO, 2018; NSWACI, 2024). The roles, responsibilities, and scope of practice for staff coordinating and delivering specialist bereavement counseling are explicitly outlined and detailed in their position descriptions (NSWACI, 2024). Data collection and analysis, including satisfaction surveys and complaints, must be conducted using quantitative and qualitative methods (NSWACI, 2024). Research should be conducted to inform evidence-based interventions (MHW, 2017; SGVDH, 2012).
8. Scope of Practice and Referral Policy. All staff delivering bereavement counseling and support must recognize the boundaries of their scope of practice. Non-specialists should be aware of their limitations and refer individuals to appropriate services when necessary (SGVDH, 2012). They should also utilize internal and external referral pathways when a bereaved individual requires interventions that exceed their professional scope or that of the organization (NSWACI, 2024). All healthcare professionals need to be informed about available bereavement resources at the local and national level, as well as referral mechanisms (Keegan et al., 2021).

### 3.2 Clinical guidelines for bereavement support in palliative care

We derived the following clinical guidelines from the principles explained in the previous section. These were organized in chronological order, considering the various moments of assessment and intervention since the entrance into palliative care, throughout the process of dying and death and during the bereavement trajectory.

1. Organizing support for the family caregiver. Seven guidelines were proposed for this stage.
  1.1 At the time of admission to palliative care, the patient should be informed that palliative care also provides support to the family (i.e., any significant person to the patient, including nuclear or extended family members, partners, friends, or neighbors) (Hudson et al., 2010, 2017; MDHBPC, 2015; NCCN, 2014).
  1.2 Ask the patient to identify their primary family caregiver. If the patient identifies only one caregiver, ask if another family member or friend is available to be contacted by the team and assume the role of an additional caregiver. Discuss the patient’s preferences regarding the involvement of the caregiver(s) in discussions about the care plan and document these in the patient’s clinical record (Hudson et al., 2010, 2012, 2017).
  1.3 Confirm with the caregiver(s) if they know the patient has designated them for this role. Explain a family caregiver’s typical role and responsibilities and confirm their willingness to accept this responsibility. Document this in the patient’s clinical record. Discuss any concerns the caregiver(s) may have about accepting this role, including potential conflicts with other family members (Hudson et al., 2010, 2012). Their ability and willingness to provide care should be regularly reassessed so that changes can be made to the intervention plan if necessary (Hudson et al., 2017; NHPCO, 2018).
  1.4 Discuss advance care planning with the patient and family, covering any implications related to the legal responsibilities of the caregiver(s) (Hudson et al., 2010, 2012, 2017; NCCN, 2014).
  1.5 Recognize the informal caregiver as an important source of information about the patient. Gather information about their experience as a support figure, including any information (when relevant) about the patient that may be considered important for the healthcare team’s knowledge (Hudson et al., 2010; Hudson et al., 2012, 2017).
  1.6 Explain to the caregiver(s) the services and resources the palliative care service can provide to establish realistic expectations (Hudson et al., 2010, 2012, 2017; Keegan et al., 2021; SGVDH, 2012).
  1.7 Have a dedicated care coordinator who facilitates communication, linking patients and families to necessary services and ensures continuity of care (Philip et al., 2018).
2. Assessing needs and establishing a care plan. Nine guidelines were proposed for this stage.
  2.1 Conduct a needs assessment with the caregiver(s), including dimensions of psychological, physical, social, spiritual, religious, cultural, financial health, and practical elements (Hudson et al., 2017; MDHBPC, 2015; NHPCO, 2018; NSWACI, 2024; SGVDH, 2012). Care and services should be aligned with patient/family caregiver needs according to the transition point in the illness. Care should continuously monitor patient/family caregiver needs (Philip et al., 2018).
  2.2 The assessment of the risk of bereavement should be an ongoing process, beginning at the time of the patient’s admission to palliative care and continuing for several months after the patient’s death. All team members can contribute to the assessment with complementary information (Hudson et al., 2010, 2012, 2017; MDHBPC, 2015; NSPCIT, 2014; NSWACI, 2024; PCA, 2018; SGVDH, 2012).
  2.3 The assessment of the risk of bereavement should be based on a conversational exploration of risk and protective factors (Keegan et al., 2021; NSWACI, 2024; SGVDH, 2012), along with data collection from the patient’s medical history and the development of a family genogram (GRPCC, 2016).
  2.4 The assessment can be complemented through the application of self-report instruments. In addition to general measures of psychosocial distress, it is recommended to use specific measures for assessing the risk of complicated grief, including: (a) Bereavement Risk Index (BRI; Parkes and Weiss, 1983); (b) Bereavement Risk Assessment Tool (BRAT; Rose et al., 2011); (c) Bereavement Risk Inventory and Screening Questionnaire (BRISQ; Roberts et al., 2016); (d) Family Relationships Index (FRI; Moos and Moos, 1981); (e) Prolonged Grief Assessment Instrument, pre-death version [PG-12; BC Centre for Palliative Care (BCCPC) Prigerson et al., 2009; Hudson et al., 2010, 2012, 2017; GRPCC, 2016; MDHBPC, 2015; Morris and Sannes, 2020; PCA, 2018; SGVDH, 2012].
  2.5 Based on the assessment, determine, in discussion with the informal caregiver, the current status and risk of psychological impairment or prolonged grief, and plan relevant interventions (Hudson et al., 2012, 2017; GRPCC, 2016; MDHBPC, 2015; NHPCO, 2018; NSWACI, 2024; SGVDH, 2012).
  2.6 When the risk is considered moderate or high, psychological or psychiatric intervention should be suggested (Keegan et al., 2021; NCCN, 2014; NSPCIT, 2014; SGVDH, 2012; GRPCC, 2016). In case of refusal, it should be indicated that the caregiver can request this support later and ask for authorization for future contacts from the team (GRPCC, 2016; Hudson et al., 2012).
  2.7 Minors affected by the patient’s death should be identified, and a plan should be developed to address their needs (NHPCO, 2018).
  2.8 A trauma-informed approach should form the foundation of assessments and support provided. This approach acknowledges the possibility that certain elements of end-of-life care may be perceived as traumatic or may trigger past trauma. It is crucial to ensure that counseling and cultural support are offered to priority groups in culturally sensitive ways and aligned with trauma-informed principles (NSWACI, 2024).
3. Ensuring information and support for the family caregiver. For this stage, 20 guidelines are proposed.
  3.1 Arrange a family meeting or conference, including the patient (Hudson et al., 2010). Family conferences provide an opportunity to share information, plan care for the patient, ensure clear communication about caregiving roles and decisions, and understand and observe the family’s functioning and relationship dynamics (Hudson et al., 2017; Morris and Sannes, 2020).
  3.2 Provide caregivers with accurate information about the disease trajectory and what to expect, which is especially relevant at different time points in the illness trajectory, including at the time of diagnosis, following a recurrence and during the end-of-life period, including the dying process (Morris and Sannes, 2020).
  3.3 Provide practical strategies to facilitate the provision of care in managing symptoms and emotional support for the patient (GRPCC, 2016; Hudson et al., 2012; NSPCIT, 2014; SGVDH, 2012).
  3.4 Reduce barriers to communication between the family/patient by promoting the expression of needs and desires of both parties and fostering reconciliation conversations (GRPCC, 2016; NHPCO, 2018). Care should include appropriate partnership and engagement of patients and family caregivers (Philip et al., 2018).
  3.5 Encourage self-care and the management of personal and social resources (GRPCC, 2016; Hudson et al., 2012, 2017; NSPCIT, 2014; Morris and Sannes, 2020; NHPCO, 2018).
  3.6 Promote adaptation to the illness by encouraging people to identify and lean on their strengths and areas of wellness (BCCPC, 2017a, 2017b). Promote adaptive coping strategies and skills training (GRPCC, 2016; Hudson et al., 2012, 2017).
  3.7 Promote an active role for the caregiver in recognizing and controlling symptoms (GRPCC, 2016; Hudson et al., 2012, 2017).
  3.8 Facilitate the process of elaborating on the various losses inherent in the advanced disease process (GRPCC, 2016; Hudson et al., 2012, 2017).
  3.9 Reinforce and validate the role played by caregivers as co-therapists at the emotional level (GRPCC, 2016; Hudson et al., 2012, 2017).
  3.10 Promote the preservation of other roles distinct from caregiving (GRPCC, 2016; Hudson et al., 2012, 2017).
  3.11 Intervene in the conspiracy of silence, as this can generate discomfort and conflicts within the family and with healthcare professionals (GRPCC, 2016; Hudson et al., 2017).
  3.12 Resolve pending practical or emotional matters (GRPCC, 2016; Hudson et al., 2012, 2017; NHPCO, 2018).
  3.13 Normalize feelings and thoughts that may provoke guilt (GRPCC, 2016; Hudson et al., 2012, 2017).
  3.14 Develop relationships with the social support network to avoid extreme dependence on palliative care teams (GRPCC, 2016; Hudson et al., 2012, 2017).
  3.15 Facilitate the emotional expression of family members (BCCPC, 2017a, 2017b; Hudson et al., 2012, 2017; MHW, 2017; NHPCO, 2018).
  3.16 Explore fears and anticipate practical organizational aspects in case the family member may be alone at the time of death (GRPCC, 2016; Hudson et al., 2012, 2017).
  3.17 Facilitate the integration of the experience and promote the search for meaning (GRPCC, 2016; Hudson et al., 2012, 2017; NHPCO, 2018).
  3.18 Help to re-establish a greater sense of control over their reality (GRPCC, 2016; Hudson et al., 2012, 2017).
  3.19 Explore relevant existential and spiritual questions (BCCPC, 2017a, 2017b; GRPCC, 2016; Hudson et al., 2012, 2017; MHW, 2017).
  3.20 Offer caregiver support groups that create a safe place for caregivers to share their stories and seek guidance (Morris and Sannes, 2020).
4. Preparing for death. Seven guidelines are proposed for this stage.
  4.1 Facilitate the decision-making process regarding the place of death and resolution of pending issues (Hudson et al., 2017; NCCN, 2014).
  4.2 Help the caregiver(s) recognize the signs that death may be imminent and the potential implications for the patient’s care needs (BCCPC, 2017a, 2017b; GRPCC, 2016; Hudson et al., 2010, 2017; MHW, 2017; NHPCO, 2018).
  4.3 When death seems imminent, assess to what extent the caregiver(s) understand the process of dying and their degree of preparation for death (BCCPC, 2017a, 2017b; GRPCC, 2016; Hudson et al., 2010, 2012, 2017; MDHBPC, 2015; NCCN, 2014).
  4.4 Encourage planning for funeral/memorial services according to their personal preferences, cultural customs and beliefs and facilitate rituals that may help the family say goodbye to the patient (Hudson et al., 2012, 2017; MDHBPC, 2015; NHPCO, 2018).
  4.5 Confirm with the caregiver(s) the type of support they may desire in pre-death accompaniment (for example, last hours, days) or immediately after (GRPCC, 2016; Hudson et al., 2010, 2017; SGVDH, 2012).
  4.6 The interdisciplinary team chooses a means of communication with the caregiver(s) to identify short- and long-term post-death responses. It is possible to refer for bereavement support at this point (Hudson et al., 2017).
  4.7 Address practical aspects related to the will and funeral arrangements, death certification, and who should be notified (GRPCC, 2016; Hudson et al., 2017; MDHBPC, 2015; MHW, 2017; NHPCO, 2018; NSPCIT, 2014).
5. Support at the time of death. For this stage, 10 guidelines were proposed.
  1. 5.1 Interdisciplinary team members should be notified of the patient’s death promptly (Hudson et al., 2010; MHW, 2017).
  2. 5.2 When the death occurs in an institutional setting (hospital, palliative care unit, nursing home) and in the absence of family members, they should be informed of the death sensitively and clearly, including other relatives (NCCN, 2014).
  3. 5.3 Sufficient time should be allowed for the family member(s) to say goodbye to the body, alone or with team members, according to their wishes (BCCPC, 2017a, 2017b; NCCN, 2014).
  4. 5.4 The family member(s) should be asked about any wishes or spiritual, religious, or cultural rituals they wish to fulfill (NHPCO, 2018; NCCN, 2014).
  5. 5.5 Ensure culturally sensitive, respectful treatment of the body (NCCN, 2014).
  6. 5.6 Post-mortem transportation and care for the body and personal belongings should be ensured with dignity and respect for the wishes and spiritual, religious, or cultural principles (MHW, 2017; NHPCO, 2018).
  7. 5.7 Normalize responses to the loss and discuss what to expect while grieving (BCCA, 2017). When appropriate, provide concise information about the grieving process (e.g., emotions and feelings that may be experienced in the acute phase of grief). The information should focus on practical and emotional support for grief, be easy to understand, and be adjusted to age, gender, and culture (BCCPC, 2017a, 2017b; NSWACI, 2024).
  8. 5.8 When death is unexpected or occurs in a particularly traumatic way, it is important to assess the degree of trauma to inform about the risk (SGVDH, 2012).
  9. 5.9 It may be necessary to postpone the first contact if the person cannot talk to the professional. In this case, it is recommended that a new contact be made 3–6 weeks after the death. This is when family and friends’ support usually decreases, and the person begins to experience a feeling of loneliness (Hudson et al., 2010).
  10. 5.10 Offer opportunities for family caregivers to return to the hospital later and meet with the team to have questions answered (Morris and Sannes, 2020).
6. Post-mortem bereavement support. For this final stage, 14 guidelines were proposed.
  6.1 The interdisciplinary team should discuss (at a reasonable time) the quality of care provided to the patient and caregiver(s)/family, circumstances of death, and impact on the family and team (at the individual and collective level) (BCCPC, 2017a, 2017b; Hudson et al., 2010, 2017; SGVDH, 2012).
  6.2 Legitimize staff discussions about the patient’s death and create a climate of safety when sharing personal issues. Provide regular opportunities for reflection and remembering through a memorial ritual for staff (e.g., brief reading, sharing stories, moments of quiet). Identify healthcare professionals at risk for complicated grief, moral distress or compassion fatigue (NCCN, 2014).
  6.3 Develop a preliminary bereavement care plan based on the needs of the caregiver(s), the pre-death risk assessment, and the circumstances of death (e.g., unexpected or traumatic) (Hudson et al., 2010). When appropriate, this screening should be supplemented with a comprehensive, holistic assessment (BCCPC, 2017a, 2017b; NHPCO, 2018; SGVDH, 2012). It is recommended that the family’s care plan constitute an independent process after the patient’s death, and it must record the specific needs and desired frequency of contact by the bereaved (NHPCO, 2018).
  6.4 Refer for psychosocial support whenever a moderate or high risk of complicated grief is identified, especially in cases where high separation anxiety and traumatic aspects related to the circumstances of death are detected (BCCPC, 2017a, 2017b; Hudson et al., 2017; NCCN, 2014; NSPCIT, 2014; SGVDH, 2012).
  6.5 If the team does not offer level two or three support, refer to teams specialized in bereavement (BCCPC, 2017a, 2017b; Hall et al., 2012; Hudson et al., 2017; NHPCO, 2018).
  6.6 Send a bereavement letter 2 weeks after death expressing the team’s feelings/condolences. If possible, personalize the letter with specific references to the patient (Hudson et al., 2017; MDHBPC, 2015).
  6.7 Attach an information bulletin with basic information about bereavement. The bulletin should focus on the following aspects: typical manifestations and available resources for bereavement support (NHPCO, 2018).
  6.8 Six to 12 weeks after death, contact the caregiver(s) or other family members (as appropriate) to provide additional information (e.g., practical information, ways to cope with acute grief symptoms, the role of palliative care team professionals in bereavement support) and assess needs. According to the assessment, the bereavement care plan should be adapted. The assessment should include: (a) Symptoms related to grief that interfere with the person’s physical and mental health (e.g., insomnia, anxiety, worsening of pre-existing health conditions, suicidal ideation); (b) Changes in functional and social capacity; (c) Bereavement overload (multiple losses in rapid succession, including concurrent losses); (d) Level of trauma caused by death and possible trauma factors; (e) Possible dissatisfaction with the notification of death; (f) Possible incongruence between the wishes expressed by the patient and the death experience; (g) Satisfaction with current social support; (h) Verify follow-up by the family doctor or other consultation (BCCPC, 2017a, 2017b; Hudson et al., 2017; Philip et al., 2018; SGVDH, 2012).
  6.9 After 6 months, those previously identified as having a risk of complicated grief should be subjected to a formal assessment using a standardized prolonged grief disorder (PGD) diagnostic instrument (BCCPC, 2017a, 2017b; Hudson et al., 2010, 2017; MDHBPC, 2015; SGVDH, 2012) Instruments for assessing Depression and Post-traumatic Stress Disorder can also be used (NSWACI, 2024). Another recommended tool is the Adult Attitudes to Grief Scale (Sim et al., 2014), which indicates the patient’s levels of vulnerability and need for support (BCCA, 2017).
  6.10 Some people may need non-specialized support. Although they do not meet PGD criteria, they may experience difficulties coping with loss, feel isolated, or need to explore their bereavement experience outside of their social context (review the circumstances of death or aspects of the relationship). In this case, it is recommended that people benefit from the support of an untrained professional or volunteer in bereavement (Morris and Sannes, 2020).
  6.11 The service partners with community providers should develop strategies and referral pathways that support families and caregivers in preparing for a loved one’s death and coping with grief. Bereaved individuals can access counseling and support services independently or through consented referrals to appropriate regional, government, non-government, or community-based services (NSWACI, 2024; PCA, 2018).
  6.12 Specialized bereavement counseling should be suggested for a person meeting PGD criteria (BCCPC, 2017a, 2017b; NSWACI, 2024; PCA, 2018; SGVDH, 2012). Approaches may include cognitive-behavioral therapy techniques, family bereavement therapy, complicated grief treatment, acceptance and commitment therapy, trauma-focused evidence-based interventions including eye movement desensitization and reprocessing (EMDR), meaning reconstruction approaches, bereavement support groups or other evidence-based focussed psychological strategies which may include interpersonal therapy, relaxation strategies (e.g., controlled breathing, progressive muscle relaxation), skills training (e.g., problem-solving, communication), psychoeducation, narrative approaches, etc. (Morris and Sannes, 2020; NSWACI, 2024; SGVDH, 2012).
  6.13 At any time when acute distress with persistent disruption of daily life, high risk of suicide, self-harm behaviors to oneself or others, or severe symptoms of depression or other mental illness is detected, immediate referral to the mental health department should be made (Hudson et al., 2017; NSWACI, 2024; SGVDH, 2012).
  6.14 At 12 months, a telephone contact should be made to determine if it is necessary to maintain the assessment and support process. Alternatively, a birthday card can be sent with reinforcement of information about the bereavement counseling contacts in case people need professional support (BCCPC, 2017a, 2017b; SGVDH, 2012).

## 4 Discussion

Support for the family and the development of a bereavement support plan are essential indicators of quality in palliative care (Morris and Block, 2015). In this literature review, we systematized the principles and clinical guidelines that ensure best practices in bereavement support for adult family caregivers in palliative care, taking into account the different stages of assessment and intervention from the initiation of palliative care, through the dying and death process, and throughout the bereavement trajectory. The results focus mainly on primary prevention measures, including providing information and practical and emotional support throughout the bereavement journey. These clinical guidelines, considered universal, should be applied to all family members from the phase preceding the patient’s death and during the period of acute grief, regardless of the degree of risk of bereavement. Simultaneously, a systematic assessment of family members should be carried out for adequate screening and referral of the most vulnerable groups in the post-mortem bereavement period. Referral to specialized levels of bereavement support can occur at any time and depends on the presence of PGD criteria and the severity of manifestations of distress. These recommendations are consistent with NICE (2004) guidelines for a more equitable and tailored response to the individual needs of bereaved people.

The application of these recommendations requires healthcare professionals to be able to offer a sensitive and appropriate response to the needs of the bereaved person. This requires adapting the practices according, for example, to the timing of entry into palliative care. In cases of late referral to palliative care, the guidelines for care organization may not apply. Furthermore, communication should be appropriate and sensitive to individual characteristics and family dynamics. In particular, preparing for death demands skills in delivering bad news, managing expectations, and responding to intense emotions. Professionals should also accurately determine the appropriate level of intervention and assess the symptoms of PGD, thereby avoiding the risk of underdiagnosing or, conversely, pathologizing normal grief. Therefore, access to training in bereavement is a consensual principle in clinical guidelines. Additionally, providing supportive conditions, such as reflective spaces and supervision for professionals, is essential to help minimize burnout caused by the emotional toll of grief. Adequate planning of bereavement support also implies articulation with spiritual and religious support services, volunteer associations, and other hospital services (e.g., psychiatry, pediatrics), health institutions, and social solidarity institutions for timely referral and collaboration in community intervention programs. The cooperation of adequately trained and supervised volunteers plays a fundamental role in intermediate-level support (including telephone contacts, one-to-one support, and management of informal support groups) and in disseminating information and sending bereavement letters. Finally, it is essential to ensure the evaluation of procedures and results (including user satisfaction) to improve the quality of services continuously.

Moreover, while structured bereavement support and professional supervision are crucial within healthcare institutions, disparities in access to these services remain a global challenge. Ensuring adequate bereavement care requires not only internal coordination among healthcare teams but also a broader commitment to equitable palliative care. The disparity in access to palliative care and bereavement support is a critical issue worldwide, particularly for patients without cancer, the oldest old, ethnic minorities and those living in rural or deprived areas are under-represented in hospice populations (Tobin et al., 2022; Kunonga et al., 2024). While hospice settings often provide structured and compassionate end-of-life care, many patients remain in acute care facilities due to the severity of their condition, resource limitations, or systemic challenges. As a result, families may not receive the same level of emotional and practical support that specialized palliative care environments offer, which can deeply impact their grieving process (Saunders et al., 2025). This highlights the need for global efforts to strengthen palliative care services and ensure that bereavement-sensitive policies extend across all healthcare settings, providing families with compassionate support regardless of where their loved one passes away.

In addition, bereavement care is often underfunded, leading to limitations in the effective application of bereavement care guidelines and, consequently, inadequate support for grieving families (Breen et al., 2014; Lichtenthal et al., 2024). Economic investments are essential for integrating bereavement services into healthcare systems to address this gap, particularly in resource-constrained settings (Lichtenthal et al., 2024). Evidence suggests that targeted funding can lead to improved outcomes. For instance, the Bupa Palliative Care Choices Program demonstrated that investments in end-of-life care enhance patient satisfaction and reduce costs by supporting care aligned with patients’ preferences (Cross et al., 2020). The quality and implementation of bereavement support programs are primarily shaped by healthcare systems’ financial and operational models. These models influence how resources are allocated, services are delivered, and the degree of prioritization given to bereavement care. To ensure minimum standards of bereavement support, it is recommended that healthcare teams develop realistic programs that include the following key elements: (1) providing dignified end-of-life care to reduce the risk of trauma for family members; (2) systematically assessing the risk of prolonged grief disorder (PGD); (3) conducting at least one follow-up contact after death to share information about available bereavement support resources (Lichtenthal, 2018).

The quality appraisal of guidelines, using AGREE II, corroborates previous findings (Kent et al., 2020), suggesting that bereavement care practice standards succeed in defining their scope, engaging stakeholders, and presenting clear recommendations. However, they present limitations in rigorously developing evidence-based content, addressing practical applicability, and demonstrating editorial independence. These results advocate for improved transparency, stronger connections between evidence and recommendations, and the inclusion of auditing mechanisms to ensure consistent quality.

The present work has limitations that should be taken into consideration. Firstly, although we recognize the need to adapt practices to users’ preferences, the clinical recommendations from this review are still not sensitive to differences in terms of the individual’s age, the nature of death, or cultural and religious diversity. The exclusion of documents based on language prevented access to a larger number of documents that would eventually reflect other cultural realities. Besides, since most guidelines are published in gray literature, it is possible that some were not included in this analysis. Gray literature, which includes reports, policy documents, and guidelines not formally published in peer-reviewed journals, often presents challenges in terms of accessibility, retrieval, and comprehensive indexing in traditional databases. Many of these documents are scattered across institutional websites, governmental agencies, and professional organizations, making systematic identification difficult. As a result, it is possible that relevant guidelines from other countries were not captured in our search. Moreover, many recommendations referring to end-of-life care do not specifically address bereavement care or, conversely, they are directed at the general bereaved population; in both cases, they were excluded from this research. This gap highlights the need for more targeted guidelines that explicitly consider the psychological and social needs of individuals experiencing grief in palliative care settings.

Another limitation of this study is that the quality appraisal was conducted after data synthesis, meaning that the quality of the guidelines was not considered during the initial stages, such as the selection of studies. Nevertheless, including lower-rated guidelines was still necessary to comprehensively overview the available literature. Also, including the quantitative analysis added an extra layer of insight, which allowed the presentation of results with a clear understanding of the strengths and weaknesses of the guidelines included in the review. For future studies, it is recommended that quality appraisal be integrated earlier in the process, particularly during the selection and data mapping phases, so that guidelines’ quality can be more effectively considered in both the inclusion of studies and the interpretation of findings.

Nevertheless, the results presented here have evident implications for clinical practice and health policies by highlighting the need to develop programs that cover different levels of bereavement support tailored to the individual needs of people. In addition to universal intervention measures of support and information, continuous assessment of symptoms of PGD and general distress should be ensured for a more appropriate and timely referral to specialized support services. On the other hand, the quality of services should be guaranteed through measures to promote training, support for professionals, and research on services. In particular, training should focus on risk assessment, diagnosis of PGD, and intervention skills appropriate to the level of intervention.

To develop evidence-based recommendations, future research should prioritize collaboration with users, including families and caregivers. This partnership is essential for adapting clinical practices in palliative care to better address their real needs, ultimately enhancing the quality of care and support. Intervention programs should focus on aspects considered helpful by the people seeking help. This calls for more inclusive, user-informed research to improve guidelines and ensure they are both evidence-based and practical (Aoun et al., 2017). More research is also needed to explore the uptake, implementation, and effectiveness of existing clinical guidelines for bereavement support in palliative care (Keegan et al., 2021). It requires a better understanding of how these guidelines are being adopted by healthcare professionals and their impact on the quality of care provided to bereaved family caregivers. Furthermore, little is known about the mechanisms of intervention that prove effective in supporting bereavement (Johannsen et al., 2019). Evidence-based guidelines should be developed directed at people with complex support needs in bereavement (with symptoms of PGD). Specifically, more robust randomized controlled trials are necessary to confirm the effectiveness of bereavement support programs, leading to evidence-based guidelines targeted at tertiary intervention.

## 5 Conclusion

This scoping review defines the international principles and clinical guidelines that should guide best practices in supporting adult family members through the grief process in palliative care. The implementation of these guidelines allows for the standardization of assessment and intervention procedures in bereavement support, with a view to the continuous improvement of the quality of services and greater effectiveness in responding to the needs of family members accompanied in palliative care.
